# Rapid, sensitive, and visible RPA-LFD assay for BoHV-1 and BoHV-5

**DOI:** 10.1128/spectrum.00895-24

**Published:** 2025-01-27

**Authors:** Yue Wu, Wenci Zhang, Chenyang Yi, Kuijing He, Changmin Hu, Guishan Ye, Anding Zhang

**Affiliations:** 1 National Key Laboratory of Agricultural Microbiology, Hubei Hongshan Laboratory, College of Veterinary Medicine, Huazhong Agricultural University, Wuhan, Hubei, China; 2 Key Laboratory of Preventive Veterinary Medicine in Hubei Province, The Cooperative Innovation Center for Sustainable Pig Production, Wuhan, Hubei, China; 3 Key Laboratory of Development of Veterinary Diagnostic Products, Ministry of Agriculture of the People’s Republic of China, Wuhan, Hubei, China; 4 International Research Center for Animal Disease, Ministry of Science and Technology of the People’s Republic of China, Wuhan, Hubei, China; 5 Guangdong Provincial Key Laboratory of Research on the Technology of Pig-breeding and Pig-disease Prevention, Guangzhou, Guangdong, China; Universidade Federal do Rio de Janeiro, Rio de Janeiro, Brazil

**Keywords:** BoHV, RPA-LFD, diagnostics

## Abstract

Bovine herpesvirus (BoHV) infection poses a significant threat to the healthy development of the cattle industry. BoHV-1 primarily causes infectious bovine rhinotracheitis, while BoHV-5 is associated with bovine necrotic meningoencephalitis. These two pathogens not only exhibit a high correlation in antigenicity and genetic background but, more importantly, can establish latent infections within the bovine ganglion. Therefore, establishing a rapid and convenient pathogen detection method for BoHV-1 and BoHV-5 is crucial for early diagnosis, epidemic monitoring, and minimizing economic losses. In this study, utilizing the conserved sequences of BoHV-1 and BoHV-5 as targets, we developed a rapid and visually accessible recombinase polymerase amplification combined with lateral flow dipstick (RPA-LFD) assay suitable for on-site detection. This method enables visual detection within 30 min for clinical samples containing as little as 1 tissue culture infectious dose 50 (TCID_50_) and performs comparably to qPCR, with slightly lower sensitivity. The findings of this research offer new insights for early clinical detection and the control of BoHV-1 and BoHV-5 infections.

**IMPORTANCE:**

Bovine herpesvirus (BoHV), a significant respiratory pathogen in cattle, is widely prevalent worldwide, posing a substantial economic threat to the cattle industry. In this study, we have developed a rapid on-site detection method for BoHV, termed RPA-LFD, which can carry out pathogen detection with a sensitivity of no less than 1 TCID_50_ within 30 min. When combined with screening, isolation, and culling measures, this approach can effectively eliminate the sources of BoHV infection, providing a scientifically feasible solution for BoHV prevention and control.

## INTRODUCTION

Bovine herpesvirus (BoHV) infections cause a variety of respiratory, digestive, and reproductive syndromes in cattle, spreading through contact between herds. These diseases exhibit diverse clinical symptoms and are prevalent worldwide. The mortality rate due to bovine herpesvirus infection ranges from 20% to 80% for cattle of various breeds and ages (1, 2). Importantly, these infections significantly impact not only the health and survival of the cattle but also influence crucial aspects, such as weight gain in beef cattle, reproductive performance in breeding cattle, and milk production in dairy cows (3, 4). Bovine herpesvirus type 1 (BoHV-1) causes infectious bovine rhinotracheitis (IBR), which presents more severely in calves. BoHV-1 infections exhibit characteristics of neurotropic and latent infections, often leading to secondary bacterial infections and, in some cases, fatalities (5). BoHV-1 infections also manifest as conjunctivitis, respiratory and reproductive tract inflammation, abortion, and necrotizing meningoencephalitis. BoHV-5 generally causes sporadic infections in herds, affecting cattle of all ages and breeds and leading to fatal necrotizing meningoencephalitis with nearly 100% mortality (6). Furthermore, the bovine herpesvirus infection currently severely constrains the intensification and scale-up of to the cattle industry (7).

As such, BoHV-1 and BoHV-5 are highly deleterious herpesviruses in cattle farming (8). Given their genetic and antigenic similarities and abilities to establish lifelong latent infections in rabbits and cattle neural ganglia (9, 10), rapid and highly sensitive diagnostic techniques are urgently needed to enable cost-effective surveillance during the control and eradication of bovine herpesvirus infections. While the real-time quantitative PCR methods recommended by the People’s Republic of China standard (GB/T 27981–2011) require expensive instruments and trained technicians, which are incompatible with point-of-care testing (POCT), the simpler gold immunochromatographic rapid assay strip lacks sensitivity for highly sensitive pathogen detection (11–13). In contrast, isothermal nucleic acid amplification coupled with visual lateral flow dipstick greatly enhances nucleic acid amplification and signal detection, enabling sampling, amplification, and readout in 30 min without the need for instrumentation (14–16). This technique has been widely applied in medical testing and food inspection (17, 18).

Here, we designed specific recombinase polymerase amplification primers and probes against conserved genomic regions to develop a recombinase polymerase amplification combined with lateral flow dipstick (RPA-LFD) assay for simultaneous detection of BoHV-1 and BoHV-5. Tests of specificity, sensitivity, and reproducibility showed that this method detected limits as low as 1 tissue culture infectious dose 50 (TCID_50_) for both viruses while exhibiting no cross-reactivity with common bovine pathogens. Clinical sample testing demonstrated 100% concordance with the People’s Republic of China standard (GB/T 27981–2011). This study provides the requisite diagnostic technology and products for rapid POCT detection of bovine herpesvirus infections to support control and eradication efforts.

## MATERIALS AND METHODS

### Viruses and cells

The strains BoHV-1, BoHV-5, BVDV, BPIV-3, BRSV, BCoV, and IDV used in this study were isolated in the field and maintained in our laboratory.

BoHV-1 propagation was performed on Madin Darby Bovine Kidney cells (MDBK, National Collection of Authenticated Cell Cultures, Shanghai, China) that were cultured in Dulbecco’s Modified Eagle’s Medium (DMEM, GIBCO, Gaithersburg, MD, USA) supplemented with antibiotics (1,000 IU/L penicillin, 100 mg/L streptomycin) and 10% fetal bovine serum (FBS, GIBCO).

### DNA and RNA extraction, cDNA synthesis

Viral nucleic acid was extracted using the Virus DNA/RNA Extraction Kit 2.0 (RM401, Vazyme, Nanjing, China). After extracting nucleic acids from RNA viruses, including BVDV, BPIV-3, BRSV, BCoV, and IDV, cDNA synthesis for the respective viruses was conducted using the HiScript II 1st Strand cDNA Synthesis kit (R211-01, Vazyme).

### Virus titration by tissue culture infectious dose 50 (TCID_50_)

MDBK cells were seeded with 50,000 in each well of a 96-well cell culture plate. The following day, the culture medium was aspirated, and the cells were washed twice with PBS. The virus solution was then 10-fold diluted using maintenance medium (DMEM with 10% FBS), and subsequently, 100 µL of the virus solution at different dilutions was added to each well. Each dilution was replicated in eight wells. After inoculation, MDBK cells were cultured at 37°C in a 5% carbon dioxide incubator for 72 h. Cell cytopathic effects were observed at 72 h post-infection, and TCID_50_ was calculated using the Reed–Muench method (19).

### RPA primers and probes design

All publicly available whole genome sequences of BoHV-1 comprising 60 records and BoHV-5 with eight records were retrieved from NCBI as of 1 March 2024. A multi-sequence alignment and homology analysis of the gE gene were performed using MEGA 7.0. Conserved regions within the gE gene were then chosen for the subsequent design of primers and LF probes. According to the TwistAmp reaction kit manuals (TwistDx Limited, Cambridge, United Kingdom), three pairs of forward and reverse primers targeting conserved regions of the gE gene were designed, along with two LF probes (Table 1). Oligonucleotide primers and probes in this study were synthesized by Tsingke Biotech (Beijing Tsingke Biotech Co., Ltd., Beijing, China).

**TABLE 1:** Primers used in this study

Primers	Sequences (5¡¯ to 3¡¯)	Products size (bp)	Location
gE-F	TGCWGCCGTACCRCTCCCACGTRTACACCC	172/98	122,223–122,394
gE-R	Biotin-TGGAAGATGCACGTCTCGTAKATGCGGAYGA		
gE-probe	FITC-TTCGACGARGCKCCSTTCTCGGCCAGCATC[dSpacer]ACTGGTAYTTCCTGCGG-C3-spacer		
2-F	CTTCTCGGCCAGCATCGACTGGTACTTC	282	122,311–122,592
2-R	AGGTCTACGCTGTTATTGGCGGGACGCAGGTG		
3-F	CCAATAACAGCGTAGACCTGGTCTTTGACGA	136/99	122,574–122,709
3-R	Biotin-CGCACCAAACGGTCCGAAGTAACGACTA		
3-probe	FITC-GGCTGCGGCCTCCGGGCTTTACGTCTTTGTG [dSpacer] TGCAGTACAACGGC-C3-spacer		

### BoHV RPA-LFD assay

Following the manual of the RPA-LFD strip amplification reagent (ADL96, D.K.Lab, Wuhan, China), for a total reaction volume of 50 µL, DNase-free water, 25 µL Buffer A, 2.5 µL Buffer B, 420 nM gE-F, 420 nM gE-R, 120 nM gE-probe, 5 µL template, and 14 mM magnesium acetate were added into the lyophilized enzyme system of the PCR tube sequentially. The mixture was incubated at 39°C for 20 min. DNA was then extracted from reaction products using phenol/chloroform and verified through 2% agarose gel electrophoresis. After being diluted 50-fold, 50 µL of the diluted RPA assay products was applied onto the sample pad of the gold immunochromatographic strip to visualize the test results. A positive result is indicated by the presence of a band at both the control line (C line) and the test line (T line), while a negative result is suggested by the absence of a band at the test line.

### Specificity of the BoHV RPA-LFD

The specificity of the BoHV RPA-LFD assay was verified among common respiratory viruses in cattle, including BoHV-1, BoHV-5, BVDV, BPIV-3, BRSV, BCoV, and IDV. DNA extraction and cDNA synthesis for the mentioned viruses were performed following the method outlined above (2.2 DNA and RNA extraction, cDNA synthesis). For each sample, 5 µL of whole genome or cDNA was used as a template, with DNase-free water serving as the negative control.

### Sensitivity of the BoHV RPA-LFD

BoHV-1 viral suspension was 10-fold diluted from 20 TCID_50_/μL to 0.02 TCID_50_/μL. Add 6% w/v Chelex 100 resin (#1421253, Bio-Rad, Hercules, CA, USA) suspension to the mentioned dilutions of the viral liquid. After thorough mixing, the solution should be incubated at 95°C for 5 min, followed by a 2 min ice bath. The samples were centrifuged at 3,000 rpm for 5 min, and 5 µL for each supernatant was used as a template to determine the sensitivity of BoHV RPA-LFD (ranging from 100 TCID_50_/reaction to 0.1 TCID_50_/reaction), with concurrent real-time quantitative PCR and performed in three replicates. The positive control comprises 200 ng of BoHV-1 genome, while DNase-free water is employed as the negative control.

### Clinical sample test

A total of 100 nasal swabs (labeled 1–100) from asymptomatic cattle and 14 nasal swabs (labeled 101–114) from cattle with respiratory symptoms were collected in 2023 and 2024, respectively, from two dairy farms in Fujian Province, China. Each of these farms house approximately 1,300 3–5-year-old multiparous cows that have been vaccinated with the inactivated IBRV vaccine. Samples were frozen and thawed once, then centrifuged at 4°C, 12,000 rpm for another 10 min. Chelex-100 and the viral solution were mixed at a w/v ratio of 100:6. The mixture was then incubated at 95°C for 5 min, rapidly cooled on ice for 2 min, and centrifuged to obtain the supernatant. Subsequently, 5 µL of each supernatant was utilized as a template in the BoHV RPA-LFD and the real-time quantitative PCR method for the detection of bovine infectious rhinotracheitis virus outlined in the People’s Republic of China standard (GB/T 27981–2011). The positive control comprises 200 ng of BoHV-1 genome, while DNase-free water serves as the negative control.

## RESULTS

### Sequence alignment, primers, and probes design

**Fig 1:**
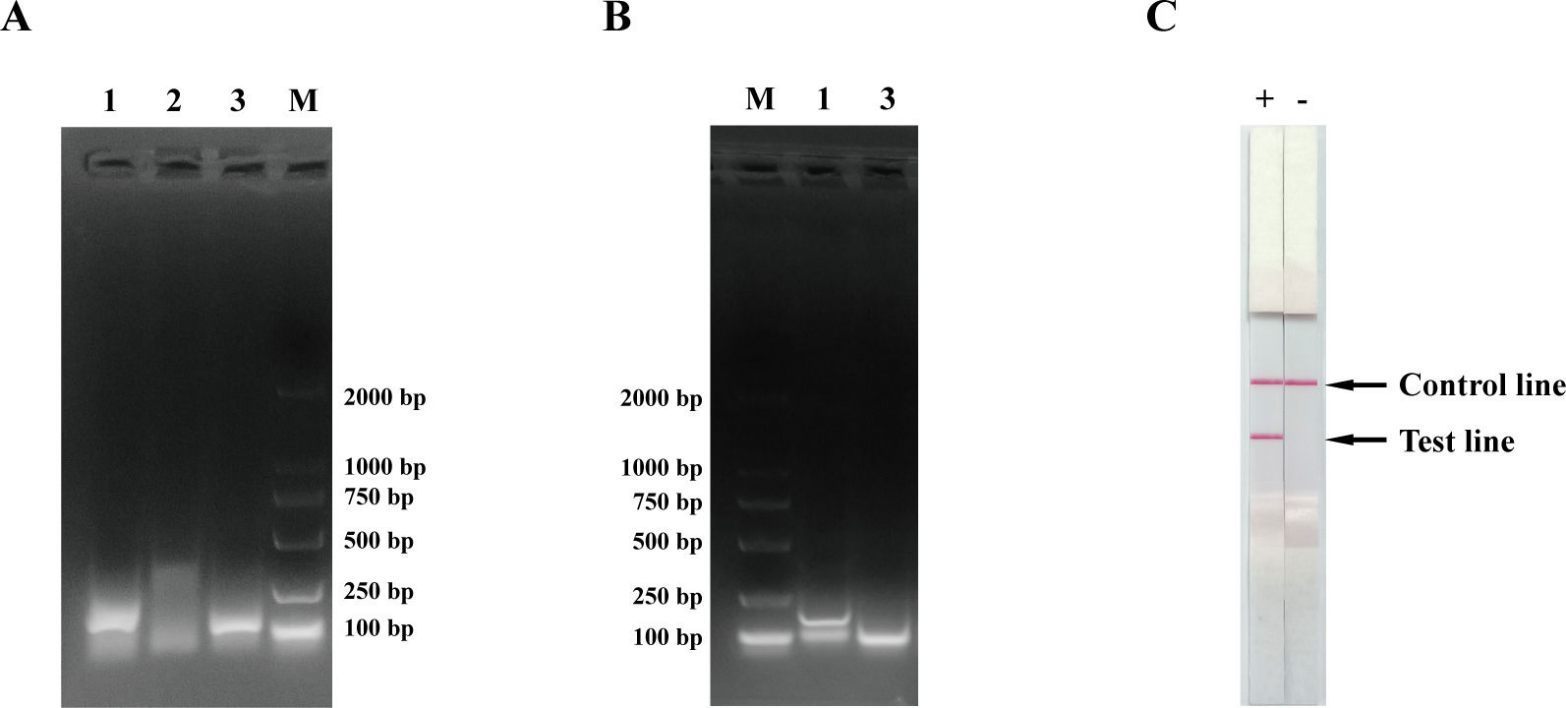
Validation of designed primers and probes for BoHV RPA-LFD assay. (**A**) Agarose gel electrophoresis of the BoHV RPA assay products using the designed primers. Lane M: molecular weight standard (DNA Marker2000). Lane 1: the amplification product obtained using primers gE-F/gE-R, with the expected band size of 172 bp. Lane 2: the amplification product obtained using primers 2F/2R, with the expected band size of 282 bp. Lane 3: the amplification product obtained using primers 3F/3R, with the expected band size of 136 bp. (**B**) Agarose gel electrophoresis of the BoHV RPA assay products using the designed primers and probes. M: molecular weight standard (DNA Marker2000). Lane 1: the amplification product obtained using primers gE-F/ gE-R and gE-probe, with the expected band size of 172 and 98 bp. Lane 3: the amplification product obtained using primers 3-F/3R and 3-probe, with the expected band size of 136 and 99 bp. (**C**) BoHV RPA-LFD analysis conducted with optimal designed primers and probe pairs gE-F/gE-R/gE-probe. Lanes + and – were conducted with templates from BoHV-1 and DNase-free water, respectively.

All publicly available BoHV-1 and BoHV-5 whole genome sequences in NCBI were first aligned and analyzed to identify conserved regions within the gE gene (Fig. 1). Agarose gel electrophoresis was performed to evaluate amplification efficiency for the three primer pairs using BoHV-1 genomic DNA extracts as template. Notably, gE-F/gE-R and 3-F/3R specifically amplified gE segment (Fig. 1A). To further test amplification efficacy of primer pairs gE-F/gE-R and 3-F/3R, LF probes were introduced into the RPA reaction system. Only gE-F/gE-R/gE-probe generated two bands, representing gE-F/gE-R amplification (172 bp) and gE-R/gE-probe amplification (98 bp) products (Fig. 1B). 3-F/3R/3-probe showed no effective amplification with the corresponding probe. Additionally, RPA-LFD with 3-F/3R/3-probe using BoHV-1 genomic DNA as template showed a positive result with presence of a band at both the C and T lines, while absence of the T line in the negative control (Fig. 1C). Thus, gE-F/gE-R/gE-probe was ultimately selected for further optimization of the BoHV RPA-LFD assay.

### Optimization of reaction conditions for the BoHV RPA-LFD assay

**Fig 2:**
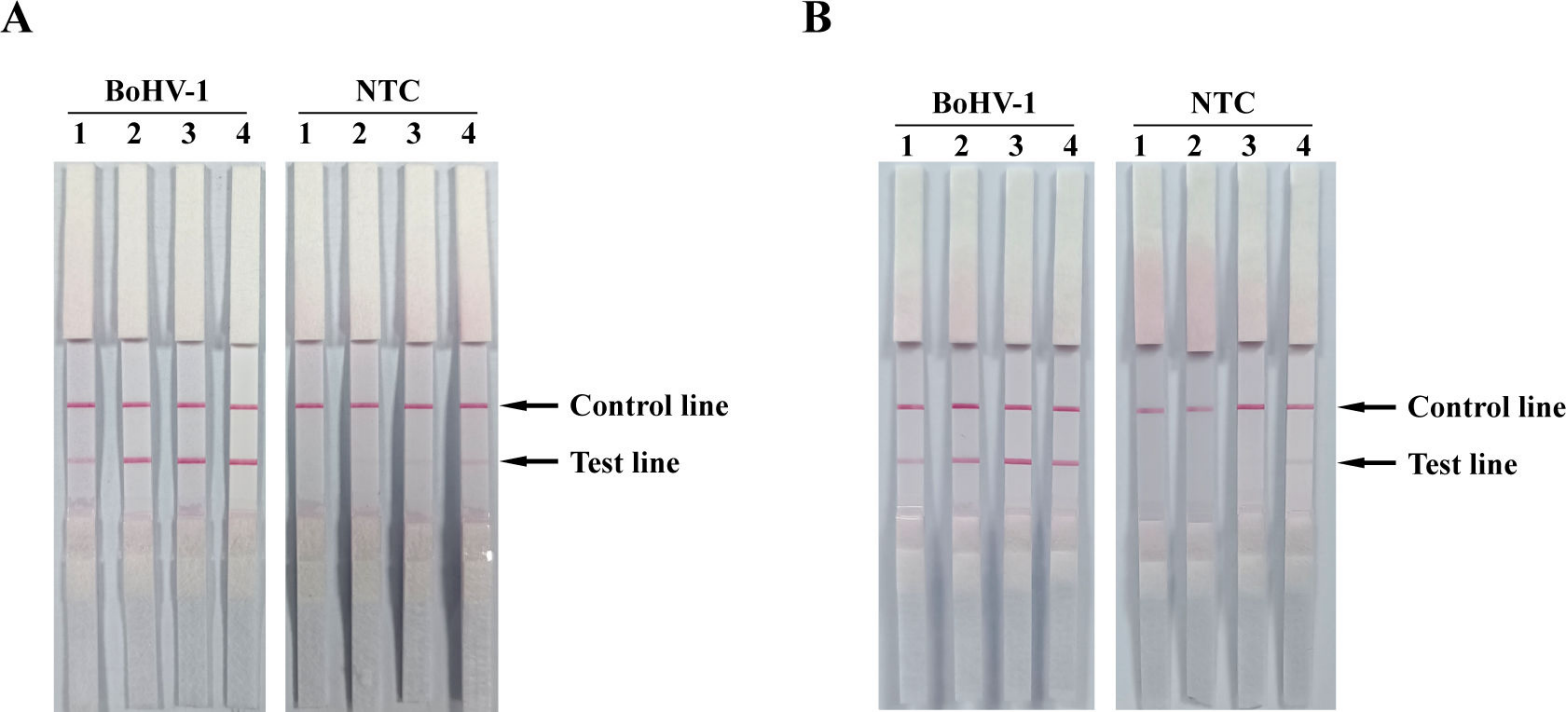
BoHV RPA-LFD assay conditions and optimization. (**A**) Temperature optimization for RPA reaction. The RPA reaction was carried out at 37°C, 39°C, 41°C, and 43°C (Lanes 1–4). (**B**) Time optimization for RPA reaction. The RPA reaction was conducted for 10, 15, 20, and 30 min (Lanes 1–4). BoHV and NTC was conducted with templates from BoHV-1 genome and DNase-free water, respectively.

After identifying optimal primer and probe combinations, we optimized RPA reaction conditions. Due to the enzyme in RPA characteristic, the ideal RPA reaction temperature range is 37°C–42°C. Thus, experiments were conducted at 37°C, 39°C, 41°C, and 43°C. The results indicated that under reaction temperatures ranging from 37 to 43°C, the BoHV-gE RPA reactions consistently achieve efficient amplification. However, at 41°C and 43°C reaction conditions, faint bands were observed in the negative control group’s test line (Fig. 2A), suggesting the occurrence of non-specific amplification at these temperatures. Therefore, the optimal reaction temperature for the BoHV RPA-LFD assay was set at 39°C. Similarly, an exploration and optimization of reaction time revealed that when the reaction time exceeded 20 min, non-specific reactions occur in the negative controls (Fig. 2B). Consequently, the optimal reaction time for the BoHV RPA-LFD assay was established as 20 min.

### Specificity of the BoHV RPA-LFD assay

**Fig 3:**
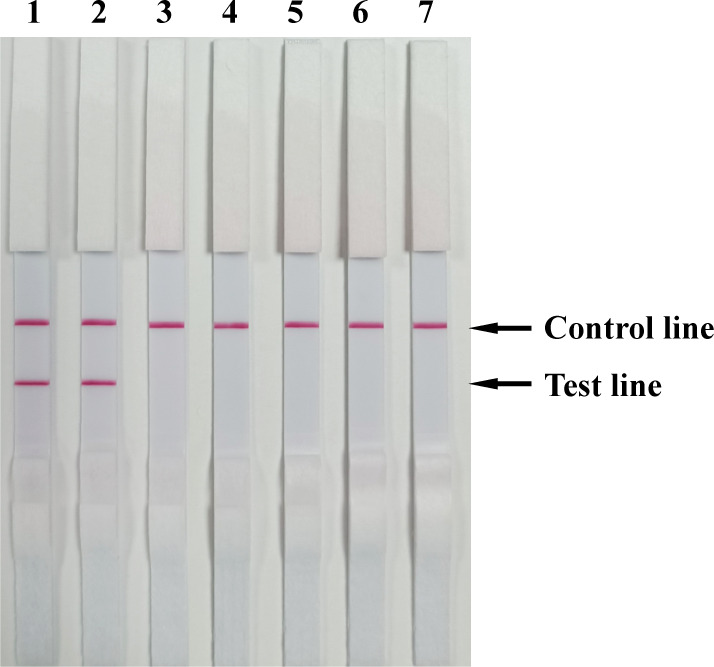
Specificity of the BoHV RPA-LFD assay. The specificity of the RPA-LFD assay was assessed for other viral pathogen genome or genome cDNA of cattle that present similarly in the clinic. Lanes 1–7: BoHV-1, BoHV-5, BVDV, BPIV-3, BRSV, BCoV, and IDV, respectively.

To assess the specificity of the established BoHV RPA-LFD assay in this study, genomic extracts from bovine parainfluenza virus type 3 (BPIV-3), bovine respiratory syncytial virus (BRSV), bovine viral diarrhea virus (BVDV), influenza D virus (IDV), and bovine coronavirus (BCoV) were reverse transcribed into cDNA and tested under the optimized BoHV RPA-LFD assay conditions (39°C, 20 min). Only templates from BoHV samples resulted in a distinct T line, while no T line reaction was observed for the other pathogens (Fig. 3). These results demonstrate the high specificity of the developed BoHV detection method, with no cross-reactivity to other bovine pathogens.

### Sensitivity of the BoHV RPA-LFD assay

**Fig 4:**
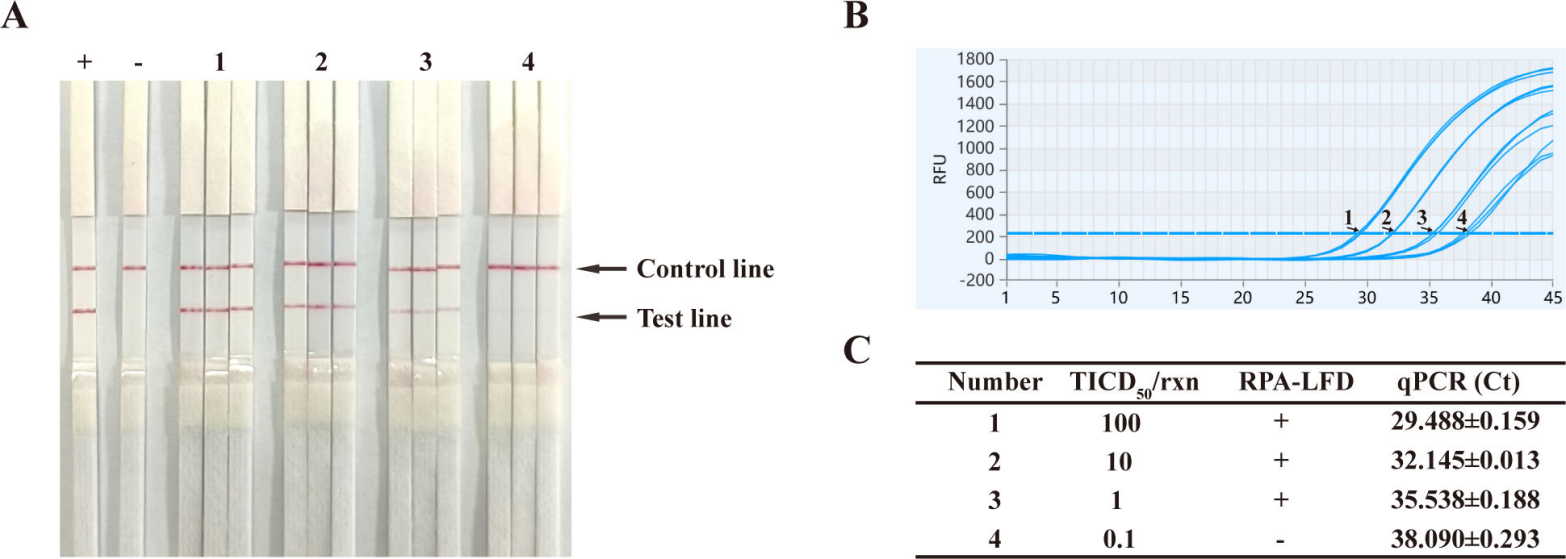
Sensitivity of the BoHV RPA-LFD assay. The viral template was serially diluted 10-fold to achieve concentrations of 100 TCID_50_, 10 TCID_50_, 1 TCID_50_, and 0.1 TCID_50_ per reaction (Lanes 1–4). Lanes labeled + and − were used as controls, with the BoHV-1 template and DNase-free water, respectively. The BoHV RPA-LFD assay (**A**) and quantitative PCR assay (**B**) were performed in triplicate for each viral template, and the detection results were compared in panel (**C**).

To determine the sensitivity of the BoHV RPA-LFD assay, the viral solution was processed with Chelex 100 resin, followed by 10-fold dilution. The reaction mixtures contained 100 TCID_50_, 10 TCID_50_, 1 TCID_50_, and 0.1 TCID_50_ viral load, respectively. The BoHV RPA-LFD assay, with the control line visible in all tests, successfully detected viral loads from 100 TCID_50_ down to 1 TCID_50_, showing sensitivity equivalent to Ct values of approximately 36 (Fig. 4).

### Detection of clinical samples

**Fig 5:**
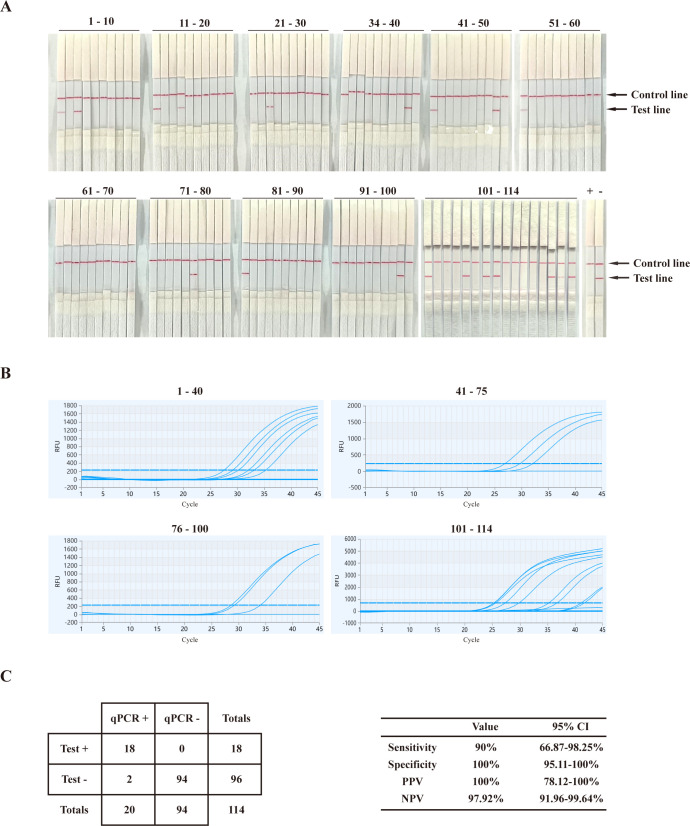
Applicability of the BoHV RPA-LFD assay. The BoHV RPA-LFD assay (**A**) and real-time quantitative PCR assay (**B**) for detection of clinical nasal swabs. Lanes+ and − used BoHV-1 genome and DNase-free water, respectively. Lanes 1–114 used the 114 field samples. Test performances (**C**) were presented as 2 × 2 tables. Positive predictive values (PPV) and negative predictive values (NPV) were calculated based on an assumed dengue prevalence of 17.54%. (Raw data are available in Table S1 in the supplemental material.).

To further evaluate the detection performance of the BoHV RPA-LFD assay, 114 bovine nasal swab samples were collected from two dairy farms in Fujian Province, China. These samples were first tested using qPCR to determine their positive (*n* = 20) and negative (*n* = 94) statuses, followed by testing with the BoHV RPA-LFD assay, including both negative and positive controls to ensure accuracy. The BoHV RPA-LFD assay effectively detected positive clinical samples, demonstrating a specificity of 100% (confidence interval [CI], 95.11%–100% [94/94 nasal swab samples]), and a sensitivity of 90% (CI, 66.87–98.25% [18/20 nasal swab samples]) (Fig. 5C). The results revealed that 18 of 114 samples tested positive on the strips, while sample NO.113 showed weak positivity (Fig. 5A). Meanwhile, these 114 clinical samples underwent real-time quantitative PCR, and the results (Fig. 5B) indicated that, among the positive samples, only samples NO.110 and NO.111 had Ct values greater than 38 (Table S1), while the Ct values for the remaining positive samples were all below 38. This outcome suggested that the BoHV RPA-LFD assay established in our study exhibits sensitivity comparable to real-time quantitative PCR in clinical testing, demonstrating excellent sensitivity and simplicity.

## DISCUSSION

The bovine respiratory disease complex (BRDC) has long been a predominant issue in cattle farming worldwide, posing a major challenge to the healthy development of the cattle industry (20). Bovine herpesvirus is the principal causative agent of BRDC, severely impacting cattle health (21). BoHV-1 and BoHV-5 share high genetic, antigenic, and clinical similarities, with respiratory disease and encephalitis as their primary manifestations (22). Importantly, their distribution patterns within infected cattle closely resemble each other, facilitating extensive colonization and replication in the upper respiratory tract (23). Considering the widespread dissemination of BoHV-1 and increasing prevalence of BoHV-5 (24, 25), the establishment of rapid and effective pathogen detection methods for both BoHV-1 and BoHV-5 is imperative for the early screening and control of bovine herpesvirus infections.

In this study, an RPA-LFD assay was developed for the rapid detection of BoHV. Our results indicated that the established detection method not only encompassed both BoHV-1 and BoHV-5, but also achieved a sensitivity of at least 1 TCID_50_ per reaction when amplifying the BoHV viral solution directly after preprocessing. In comparison with the real-time quantitative PCR method commonly used in laboratory diagnostics, the sensitivity of the BoHV RPA-LFD assay was essentially equivalent to Ct values of approximately 36, displaying no occurrence non-specific reactions. A previous study reported an RPA-based method targeting the BoHV-1 TK gene, combined with a closed vertical flow visual band (VF) technique, with detection limits of 10.9 TCID_50_ per reaction and 38.8 copies/μL for BoHV-1 virus and the positive plasmid containing the target fragment, respectively (16). In contrast, the method developed in this study demonstrates a significantly lower detection limit of 1 TCID_50_ for the virus, representing an approximately 10-fold increase in sensitivity. Additionally, our method targets the conserved region of the gE gene shared by both BoHV-1 and BoHV-5, enabling simultaneous detection of both viruses, as well as differentiation between wild-type BoHV infections and gE gene-deleted vaccine strain infections in cattle. This unquestionably harbors significant potential for the detection of clinical samples. Furthermore, RPA demonstrates significant tolerance to common PCR inhibitors and has been successfully applied to nucleic acids extracted from a variety of sample types, including nasal swabs, serum (26), blood, feces, tissues (15), and milk (27). Additionally, the characteristics of RPA components being amenable to lyophilization for convenient transport and storage were advantageous for meeting the rapid detection demands at farms (28, 29).

BoHV-1 and BoHV-5 have been reported to induce infections and outbreaks on a global scale. To date, some developed countries have achieved BoHV-1 eradication by marker vaccines; however, in domestic contexts, the control strategy relies mainly on surveillance-culling, posing tremendous challenges including the numerous inputs of the human and material resources (30). The establishment of such a rapid and portable detection method in this study undoubtedly emerges as a key component in the prevention and eradication efforts against BoHV. Furthermore, genetically engineered vaccines with gE gene deletion of herpesviruses have gained widespread acceptance internationally (31, 32). They play a pivotal role in the eradication process of animal epidemic disease (33, 34). Therefore, the RPA rapid detection method designed for the gE genes of BoHV-1 and BoHV-5 in this study can also serve as a crucial tool to distinguish the wild-type infections from vaccine–strain infections during the eradication process in domestic settings. This offers a robust mechanism for the prevention and eradication of BoHV-1 and BoHV-5.

## Data Availability

The data that support the findings of this study are available upon reasonable request.

## SUPPLEMENTAL MATERIAL

Figure S1 spectrum.00895-24-s0001.docx Nucleotide sequence alignment of the amplified fragment of BoHV-1 and BoHV-5 gE genes.

Table S1 spectrum.00895-24-s0002.csv BoHV detection in nasal swabs with real-time quantitative PCR assay and BoHV RPA-LFD assay.

ASM does not own the copyrights to Supplemental Material that may be linked to, or accessed through, an article. The authors have granted ASM a non-exclusive, world-wide license to publish the Supplemental Material files. Please contact the corresponding author directly for reuse.
