# Supplementary material for: Rapid, sensitive, and visible RPA-LFD assay for BoHV-1 and BoHV-5
Source: Microbiol Spectr. 2025 Jan 27;13(3):e00895-24. doi: 10.1128/spectrum.00895-24 (PMC11878031; doi:10.1128/spectrum.00895-24)
Supplement: Figure S1 — Nucleotide sequence alignment of the amplified fragment of BoHV-1 and BoHV-5 gE genes. [file spectrum.00895-24-s0001.docx]

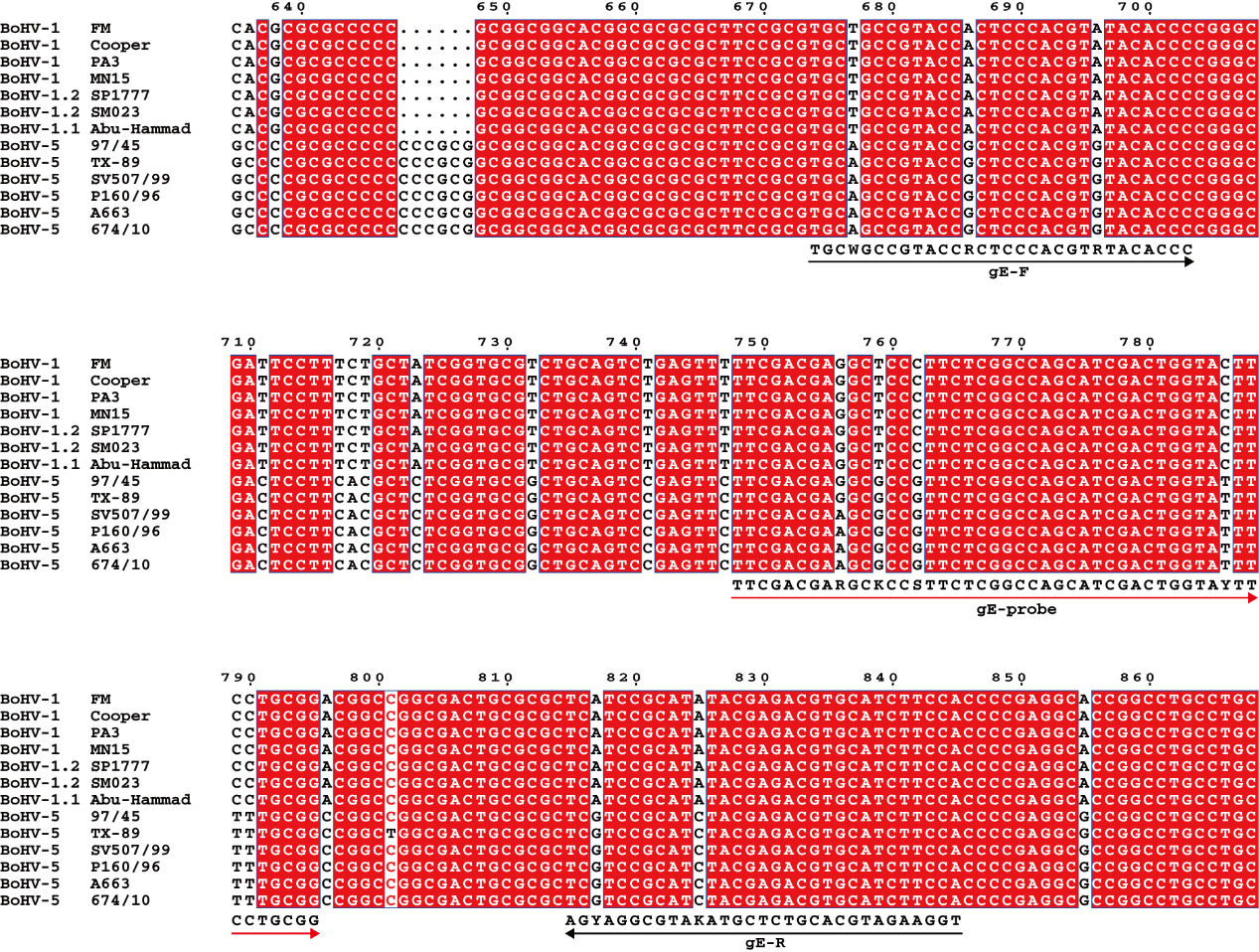


**Supplementary Figure 1 Nucleotide sequence alignment of the amplified fragment of BoHV-1 and BoHV-5 gE genes.** The sequences of forward primer and the reverse primer are showed below the sequences, as black arrow, and the LF probe was showed as red arrow.
